# Intranasal Oxymetazoline and Xylometazoline Use in Patients With Deviated Nasal Septum: A Cross‐Sectional Telephone Survey

**DOI:** 10.1002/oto2.70216

**Published:** 2026-03-09

**Authors:** Marcin Masalski, Jakub Kurasz, Aleksandra Kosiorowska, Aleksander Mateja, Krzysztof Morawski

**Affiliations:** ^1^ University of Opole Institute of Medical Sciences Opole Poland; ^2^ Department of Otolaryngology University Clinical Hospital of Opole Opole Poland; ^3^ University of Opole, Institute of Medical Sciences Student Research Group in Otorhinolaryngology Opole Poland

**Keywords:** deviated nasal septum, intranasal decongestants, nasal congestion, nasal spray addiction, oxymetazoline, rebound nasal congestion, xylometazoline

## Abstract

**Objective:**

To assess the pattern of oxymetazoline and xylometazoline (OXM) use in patients with deviated nasal septum (DNS).

**Study design:**

A retrospective Computer‐Assisted Telephone Interviewing survey.

**Setting:**

Patients who underwent septoplasty in University Clinical Hospital in Opole, Poland.

**Methods:**

A retrospective Computer‐Assisted Telephone Interviewing (CATI) survey was conducted among patients who underwent septoplasty between 2018 and 2024. The questionnaire included inquiries about the frequency of OXM use, awareness of the consequences of long‐term OXM application, and alternative treatment options, all prior to septoplasty.

**Results:**

The study was conducted on 159 of 305 (52.1%) patients identified in the hospital database. Approximately one‐third of respondents (55 of 159, 34.6%) denied using OXM, about one‐third had used OXM for no more than 7 days (52 of 159, 32.7%), and the remaining one‐third (52 of 159, 32.7%) admitted to OXM overuse. Lack of awareness of the consequences of chronic OXM use was associated with a 6.5‐fold increased risk of addiction (OR = 6.5, 95% CI: 1.2‐33.6, *P* = .02). Furthermore, 16 of 52 (30.8%) respondents were unaware of intranasal steroid therapy in the preoperative period.

**Conclusions:**

DNS may increase the risk of OXM dependence; however, further controlled studies are needed. Increasing awareness about the risks of OXM overuse substantially reduces this risk.

A deviated nasal septum (DNS) is a common cause of nasal obstruction in patients seeking consultation with an ENT specialist.^1^ This condition leads the patient to adopt non‐physiological mouth breathing, which may contribute to an increased frequency of nasal infections, recurrent sinusitis, and even the development of sleep apnea.^2^ Impaired nasal breathing and cleansing functions due to a deviated nasal septum has been shown to increase the risk of sinus and upper respiratory tract infections.^3^

Deviation of the nasal septum has been demonstrated to promote chronic nasal infections, rhinosinusitis, and rhinorrhea.^4^, ^5^ When experiencing such symptoms, patients typically resort to readily available nasal decongestants.^6^

The most commonly used nasal decongestants are imidazoline derivatives, oxymetazoline, and xylometazoline (OXM).^7^ The mechanism of action of OXM involves the stimulation of α2‐adrenergic receptors, leading to vasoconstriction, which consequently relieves swelling, reduces congestion of the nasal mucosa, improves sinus drainage, and enhances nasal breathing.^8^, ^9^ Due to the rapid contraction of the nasal mucosa, these drugs are readily chosen by patients.^9^ In addition in many countries OXM is OTC (over‐the‐counter) and available without a prescription.

Frequent use of OXM can lead to rebound congestion (secondary edema of the nasal mucosa) from prolonged stimulation of α2‐adrenergic receptors.^9^ Episodes of mucosal ischemia following OXM use, along with subsequent compensatory mucosal congestion when OXM stops working, cause chronic mucosal edema.^10^ This has led a significant number of patients to resort to additional doses of the drug, thus creating a self‐perpetuating cycle of addiction to OXM. Rebound congestion symptoms manifest within 3‐10 days of OXM use, although some studies indicate that they can develop as late as 30 days.^11^, ^12^, ^13^

A study of patients with the common cold revealed that 92% (937 of 1019) of them used nasal decongestants as prescribed, with a median duration of 6 days.^14^ The study found that nasal decongestants had a favorable safety profile and were highly satisfactory to patients in the OTC setting. Similarly, a study on the general population of Saudi Arabia showed that only 7.1% (103 of 1456) reported using nasal decongestants for more than two weeks.^15^ Although the general population appears to be at low risk of nasal decongestant overuse, certain groups of patients may be more susceptible. For instance, among patients with persistent allergic rhinitis, nearly half may be affected.^16^ Another group at risk of overuse may be people with DNS.

In patients diagnosed with DNS, upper respiratory tract infections are characterized by greater severity, which may lead to the frequent use of OXM.^17^ Moreover, OXM temporarily alleviates the discomforts associated with DNS, such as nasal congestion, airflow obstruction in the nasal cavities, abnormal airflow in the nasal sinuses and impairment of auditory tube patency.^8^ While the optimal treatment for DNS accompanied by persistent symptoms is nasal septal surgery, the ready availability of OXM postpones the decision for surgery and increases the risk of dependence.^18^, ^19^ A similar pattern of OXM overuse is observed in conservative DNS treatment, where non‐addictive medications, such as intranasal steroids with saline rinses, are employed.^20^ However, unlike OXM, these medications have reduced effectiveness and considerably delayed onset of action.

Considering the factors influencing the varying patterns of OXM use among DNS patients, a retrospective questionnaire‐based study was conducted. The study aimed to assess OXM consumption patterns and awareness of the risks associated with OXM overuse among patients requiring surgical intervention for DNS.

## Methods

The survey was designed as a cross‐sectional study using a Computer‐Assisted Telephone Interviewing (CATI) survey, in accordance with the Checklist for Reporting Survey Studies (CROSS) methodology.^21^ The survey was conducted among patients with DNS requiring surgical treatment at the University Clinical Hospital of Opole, who had undergone the procedure between 2018 and 2024. Patients provided responses regarding their condition prior to the surgery. The study was approved as exempt from full review by the Bioethics Committee of the University of Opole (L.dz. 83800.0050.6.2025).

### Material

In this study, a convenience sampling approach was employed. The target population of individuals with DNS consisted of patients who underwent septoplasty at the University Clinical Hospital of Opole. Contact data for these individuals were readily accessible from the hospital's information system. The exclusion criteria included patients with significant cognitive impairments that could affect their ability to understand the questionnaire, as well as individuals who were not proficient in the language used in the questionnaire. Patients were not reimbursed for participating in the survey.

### Survey

The survey comprised 6 sections with a total of 18 questions (17 of which were closed‐ended questions, including 1 multiple‐choice question and 1 open‐ended question). The survey was prepared using Google Forms. The first section covered general information such as gender and age. The subsequent questions were taken from the Nose Obstruction Symptom Evaluation (NOSE) questionnaire, which assesses the severity of nasal obstruction symptoms.^22^, ^23^ Questions from the NOSE questionnaire were presented on a linear scale, where 0 points indicated no problem at all and 4 points indicated a major problem. Further questions concerned the frequency and duration of OXM use, and awareness of OXM side effects. Patients were instructed to answer questions regarding their condition prior to septoplasty. Based on regulatory drug information, overuse of OXM was defined as its use for more than 7 consecutive days. In addition, the questionnaire included questions about the use of other medications, including nasal steroids and saline preparations. The full survey, along with its English translation, is provided in Supplement Appendix 1, available online. The survey was reviewed by experts and then tested online, including a simulated survey completion.

The survey was conducted by 4 interviewers, each of whom had undergone training to ensure consistency in providing information to patients. Particular emphasis was placed on uniformly translating unclear questions, collecting and entering data, and adhering to ethical standards to ensure the accuracy and reliability of the results. Each interviewer had a list of patient identifiers, along with their assigned telephone numbers, and patients were called at different times of the day from designated stations. During these calls, patients were informed that their participation in the survey was entirely voluntary, and that no sensitive data were collected. The interviewers then entered the interview data into a Google Forms survey. Notably, no patient completed the survey more than once, and all responses were collected during a single telephone interview.

### Statistical Analysis

The sample size was calculated assuming a prevalence of OXM overuse of 30% in the target population, a margin of error of 7.5%, and a statistical confidence level of 95%. This resulted in a required sample size of 144 participants.

Statistical analysis of the results was conducted using the Kruskal‐Wallis test and confidence interval analysis. The calculations were performed using the Matlab R2020b environment (MathWorks Inc.).

## Results

**Figure 1:**
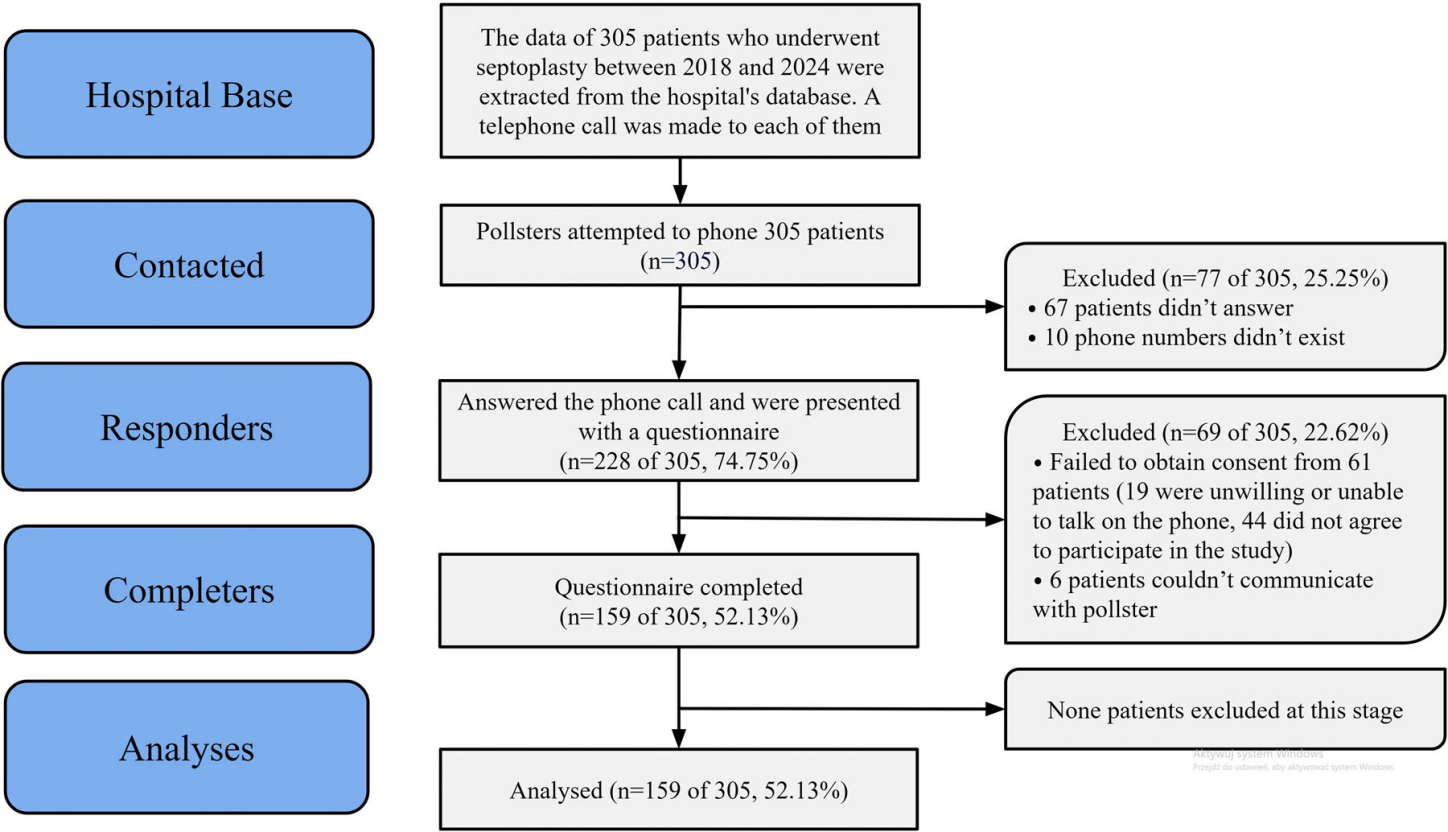
Study participant flowchart.

**Figure 2:**
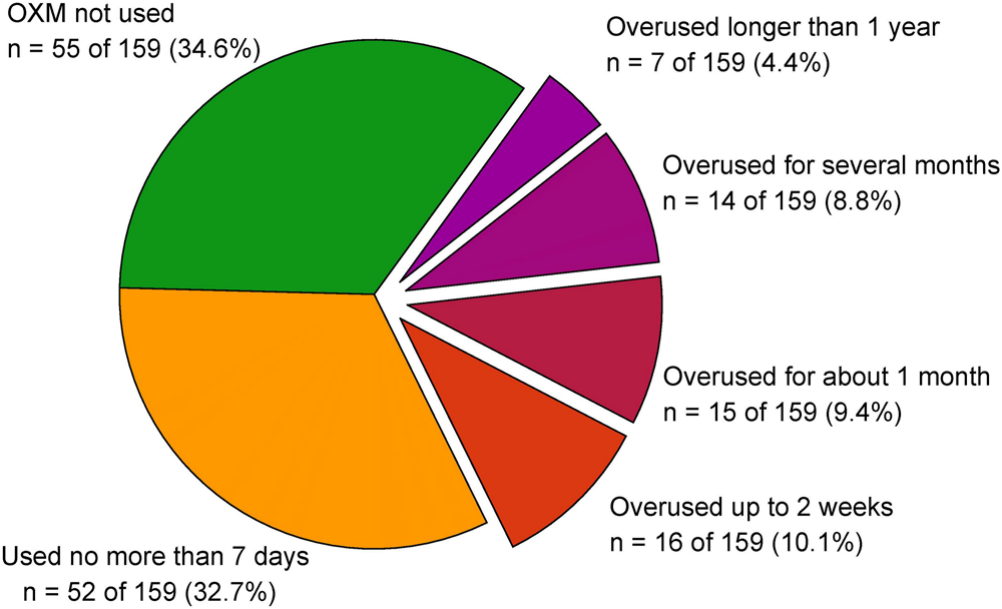
A profile of the usage of oxymetazoline and xylometazoline (OXM).

Between November 13, 2024, and December 21, 2024, attempts were made to contact 305 individuals identified in the hospital database who had undergone septoplasty between 2018 and 2024. A total of 159 of 305 (52.13%) individuals consented to participate in the survey. The remaining individuals either declined to participate (69 of 305, 22.62%) or could not be reached (77 of 305, 25.25%). The flowchart is shown in Figure 1 and the results for each question of the survey are provided in Appendix 1. The mean time from surgery to survey completion was 3.6 years (SD 2.0, range 0‐6 years), with a median of 4 years. The mean age of the respondents at the time of surgery was 39.7 years (SD 13.2, min 17, max 71). The majority of respondents were male (109 of 159, 68.6%, 95% CI: 61.3‐75.8). The average NOSE survey score was 11.3 with a standard deviation SD = 4.0. OXM was used by 104 of 159 respondents (65.4%, 95% CI: 58.0‐72.8), and 52 of 159 (32.7%, 95% CI: 25.4‐40.0) admitted to overusing OXM (Figure 2), that is, using it for more than 7 consecutive days. Among respondents who overused OXM, 21 of the 52 respondents (40.4%, 95% CI: 27.0‐53.7) reported continuous use of OXM for more than 1 month, and 22 of the 52 respondents (42.3%, 95% CI: 28.9‐55.7) reported a period of overuse longer than 3 years.

**Figure 3:**
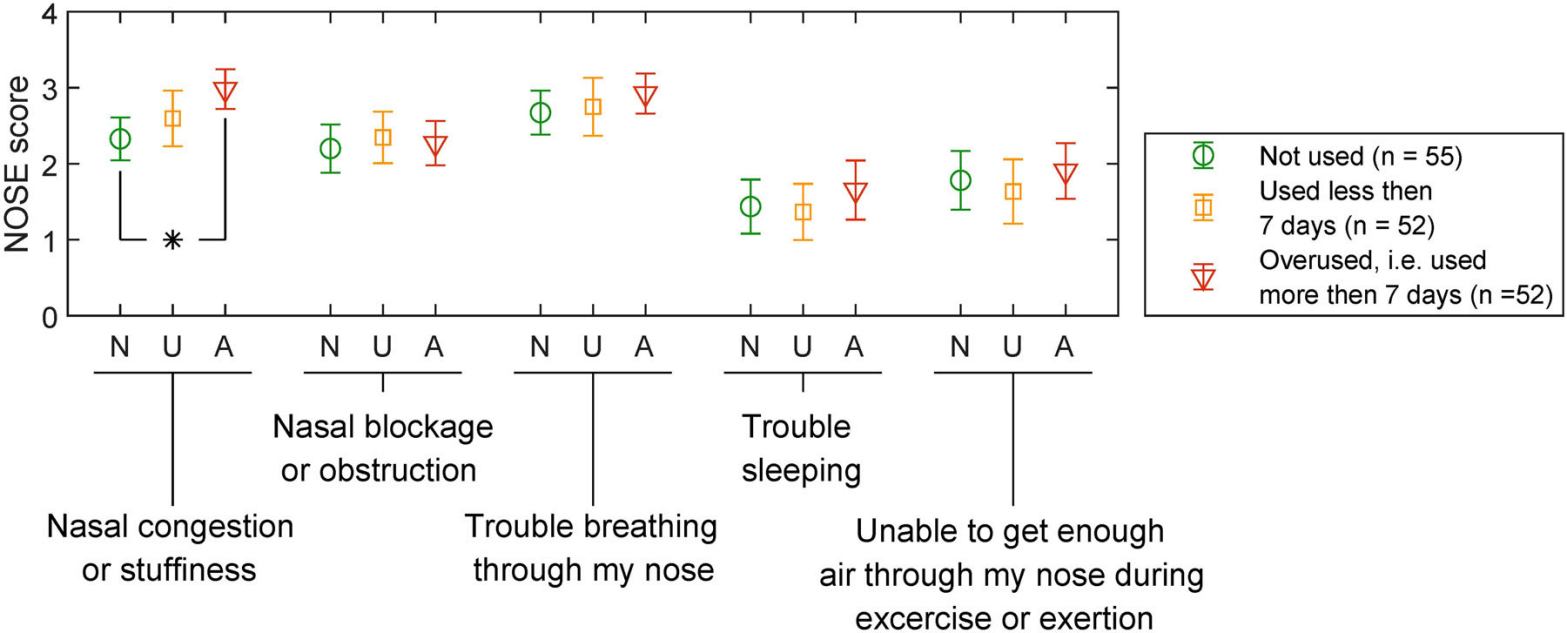
NOSE questionnaire results stratified into three groups by OXM use: nonusers, users for ≤7 consecutive days, and overusers (>7 consecutive days). Statistically significant differences (*P* < .05) are indicated by (*). NOSE, Nose Obstruction Symptom Evaluation; OXM, xylometazoline.

The Kruskal‐Wallis test was conducted on the symptoms reported in the NOSE questionnaire across three groups: nonusers, users, and overusers of OXM. A statistically significant difference was observed only for nasal congestion/stuffiness (*P* < .001, *ε*² = 0.09). Post hoc Dunn‐Sidák's test indicated significantly higher nasal obstruction scores in overusers compared to nonusers (*P* < .001). No statistically significant differences were found between the other groups at the significance level of 0.05. A comparison of the results from the NOSE questionnaire is presented in Figure 3.

The most prevalent reason for OXM use was the intention to enhance nasal breathing comfort (47 of 52 respondents, 90.4%, 95% CI: 82.4‐98.4), resulting in an inability to function without OXM in 15 of those (28.8%, 95% CI: 16.5‐41.2). Significant reductions in discomfort associated with OXM dependence were reported by 8 of 52 respondents (15.4%, 95% CI: 5.6‐25.2) following intranasal steroid use and by 12 of 52 respondents (23.1%, 95% CI: 11.6‐34.5) following nasal irrigation. Notably, 16 of the 52 respondents (30.8%, 95% CI: 18.2‐43.3) were unaware of the potential for using intranasal steroids.

**Figure 4:**
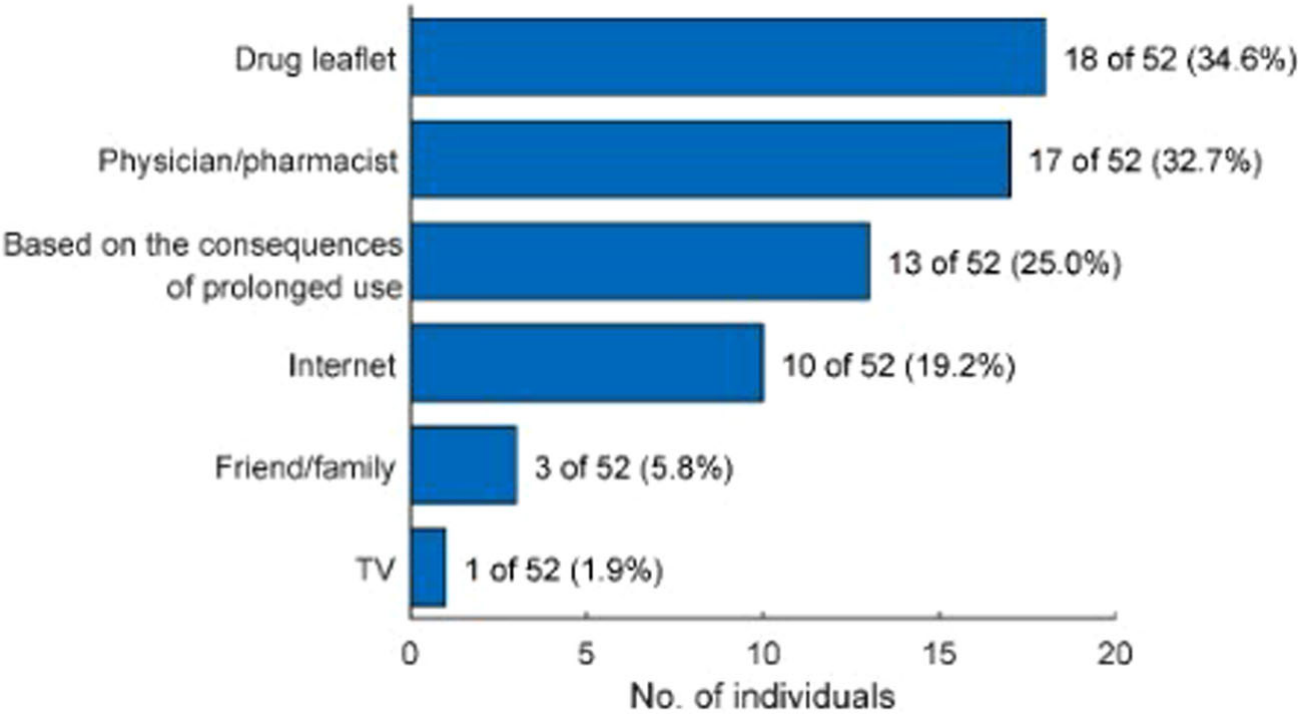
Sources of information on the adverse effects of prolonged oxymetazoline and xylometazoline (OXM) use among respondents who have used it for more than 7 consecutive days (n = 52, with multiple selection allowed).

**Figure 5:**
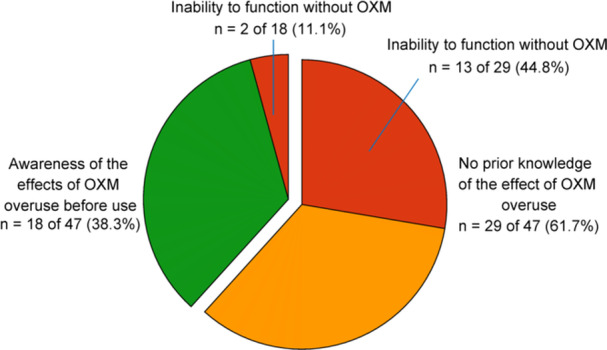
Individuals who developed dependence on oxymetazoline and xylometazoline (OXM) after using it to enhance nasal breathing comfort, stratified into two groups: those aware of the effects of OXM overuse and those unaware.

The respondents exhibited a range of awareness regarding the adverse effects associated with long‐term OXM use. Specifically, 22 of 52 respondents (42.3%, 95% CI: 28.9‐55.7) indicated awareness of these effects, while 10 of 52 respondents (19.2%, 95% CI: 8.5‐29.9) reported that they became aware of the undesirable effects only after they had manifested. The primary sources of information regarding the adverse effects of chronic OXM use cited by respondents were the drug leaflet (18 of 52, 34.6%), advice from a medical professional (17 of 52, 32.7%), and personal observation of adverse effects following OXM use (13 of 52, 25.0%) (Figure 4). Among individuals who initiated OXM use to enhance breathing comfort, those unaware of the consequences of OXM overuse had a 6.5‐fold increased odds (95% CI: 1.2‐33.6, *P* = .02) for developing functional impairment in contrast to those who were aware (Figure 5).

## Discussion

In the cohort of patients with DNS qualified for surgical intervention, approximately one‐third (55 of 159, 34.6%) denied using OXM, another third reported using OXM for no longer than 7 days (52 of 159, 32.7%), and the remaining third (52 of 159, 32.7%) admitted to OXM overuse. These findings contrast with data from studies conducted in the general population. In a study on nonallergic rhinitis, the combined prevalence of regular OXM use across the study and control groups was 8.1% (93 of 1153).^24^ Another population‐based study reported OXM use exceeding 2 weeks in 7.1% (103 of 1456), compared with 22.6% (36 of 159) in our DNS cohort.^15^ Although these comparisons are descriptive and should be interpreted with caution due to differences in study design and potential confounders, they suggest a possible signal that patients with DNS may be at increased risk of prolonged OXM use. This phenomenon may be related to more frequent and severe upper respiratory tract infections in patients with DNS, as well as the temporary alleviation of DNS‐related symptoms following OXM use.^8^, ^17^ Clinicians should therefore remain aware of the high prevalence of prolonged OXM use in DNS patients when considering its prescription.

The survey results showed that 30 of 52 individuals (57.7%) were unaware of the negative effects of OXM overuse. A lack of awareness regarding the consequences of OXM overuse significantly increases the likelihood of developing dependence on OXM, resulting in 6.5‐fold increased odds (OR = 6.5, 95% CI: 1.2‐33.6, *P* = .02) for an inability to function without OXM compared to informed individuals. This observation is particularly relevant given the widespread availability of OXM, which is OTC in most countries, and its aggressive marketing by pharmaceutical companies. However, the increased accessibility of OXM has been shown to reduce the number of medical visits, thereby alleviating the burden on healthcare systems, particularly in countries with a high prevalence of upper respiratory tract infections and DNS.^25^ Nevertheless, uncontrolled access to OXM, associated with aggressive marketing strategies, promotes OXM misuse, particularly when advertising messages provide only general information about the drug without detailed warnings about the consequences of overuse. In this context, patient education, as demonstrated in this study, plays a crucial role to reduce the risk of dependence.

The most common sources of information about the adverse effects of OXM among respondents were physicians, pharmacists, and the medication package leaflets. These findings highlight the crucial role of healthcare professionals in educating patients about the potential adverse effects of medications, and emphasizing the importance of equipping physicians and pharmacists with the necessary competencies to effectively educate patients.^26^, ^27^

A detailed analysis of the NOSE questionnaire results revealed that patients with excessive OXM use reported the highest scores for the specific question addressing the sensation of nasal obstruction or congestion. This finding indicates a strong association between OXM overuse and impaired nasal function and is in line with expectations, as OXM overuse leads to rebound nasal mucosal swelling. The study found that 15 of 47 patients (31.9%) who initially overused OXM solely to improve their nasal breathing comfort subsequently developed dependence on OXM, manifesting as secondary mucosal swelling.

The conservative management of symptoms associated with a DNS involves the administration of intranasal corticosteroids (INCS) and nasal irrigation.^4^ As demonstrated in this study, all respondents were aware of the potential for nasal irrigation, however, a significant proportion, specifically 16 of 52 respondents (30.8%), lacked knowledge regarding the availability of INCS. This finding suggests a potential gap in patient education by healthcare providers regarding INCS, which may contribute to patients' perceptions of INCS as being comparable to systemic steroid therapy. Research indicates that only 41.9% of patients perceive INCS as effective, while 27.8% consider them safe.^28^ However, the survey results highlight the effectiveness of promotional campaigns targeting nasal irrigation products.

### Limitations

The interval between septoplasty and the survey might have extended up to 6 years, during which some patients could have had difficulty accurately recalling the timing of OXM use, steroids use, or symptoms assessed in the NOSE questionnaire after this extended period.^29^ However, research indicates that the mean percentages of overestimation and underestimation for both duration and severity of health events in retrospective studies often remain comparable.^30^

The inclusion criteria for the study were limited to patients who had undergone septoplasty for DNS. Therefore, it can be expected that the severity of OXM overuse would be lower in patients with mild DNS who do not require septoplasty.

### Generalizability

The survey revealed a higher prevalence of DNS requiring surgery among males, with 31.4% of respondents identifying as female and 68.6% as male. This finding aligns with the results of studies on similar topics, which also demonstrated a comparable sex ratio among patients with DNS, with percentages ranging from 64.9% to 77.3% in favour of males.^31^, ^32^ The mean age of the respondents in the study was 39.7 years, which is consistent with findings reported in studies; according to recent research, the average age of patients undergoing septoplasty ranges from 36.24 to 43.4 years.^33^, ^34^ In both of these aspects (sex distribution and age) the study population aligns with those in other scientific studies.

The present study was conducted on a population of a Central European country, with all participants being Caucasian Europeans. This is significant in the context of DNS prevalence, as studies have shown that the larger nasal septum size in Caucasians may contribute to a greater degree of DNS.^35^ Consequently, individuals of Caucasian descent may be more susceptible to OXM overuse than other ethnic groups.

OXM nasal sprays are available without a prescription in Poland and most European countries, including Germany and France, as well as in countries with similar pharmaceutical regulations, such as the United States. Furthermore, in all of these countries, OXM is advertised in the mass media increasing its visibility among patients. Therefore, it can be expected that the findings of this study can be generalized to other populations.

## Conclusions

The prevalence of OXM overuse among patients with DNS appears to be higher than general population estimates reported in other studies. Therefore, patients with DNS should be counseled regarding the risks of OXM overuse, as appropriate guidance may significantly reduce the likelihood of dependence. A substantial number of DNS patients were unaware of the potential for intranasal steroid therapy in preoperative management, underscoring the need for educational initiatives in this area.

## Author Contributions

**Marcin Masalski**, conception and design of work, analysis of data, drafting and revising manuscript, and final manuscript approval; **Jakub Kurasz**, acquisition of data, manuscript drafting and revising, and final manuscript approval; **Aleksandra Kosiorowska**, acquisition of data, drafting and revising manuscript, and final manuscript approval; **Aleksander Mateja**, acquisition of data, manuscript revision and final manuscript approval; **Krzysztof Morawski**, revising manuscript and final manuscript approval.

## Disclosures

### Competing interests

None.

### Funding source

None.

## Supporting information

Appendix 1 Survey on the use of oxy‐ and xylometazoline and its results. Original Polish version is given in italics. ^a^The age at the time of septoplasty, calculated on the basis of current age and the year of the procedure, is: mean 39.7, median 38, min17.0, max 71.0, SD 13.2.
