# Supplementary material for: Intranasal Oxymetazoline and Xylometazoline Use in Patients With Deviated Nasal Septum: A Cross‐Sectional Telephone Survey
Source: OTO Open. 2026 Mar 9;10(1):e70216. doi: 10.1002/oto2.70216 (PMC12969495; doi:10.1002/oto2.70216)
Supplement: Supplementary file 1 — Appendix 1 Survey on the use of oxy‐ and xylometazoline and its results. Original Polish version is given in italics. aThe age at the time of septoplasty, calculated on the basis of current age and the year of the procedure, is: mean 39.7, median 38, min17.0, max 71.0, SD 13.2. [file OTO2-10-e70216-s001.docx]

| Answer | Results |
| --- | --- |
| Sex *(płeć)* | |
| Male  *(Mężczyzna)* | 109 of 159 (68.6%) |
| Female  *(Kobieta)* | 50 of 159 (31.4%) |
| Current age *(Aktualny wiek)^a^* | |
| Age  *(Wiek)* | mean 43.3, median 42, min 19, max 75, SD 13.1 (n=159) |
| Year of septoplasty *(Rok operacji septoplastyki)* | |
| 2018 | 40 of 159 (25.2%) |
| 2019 | 29 of 159 (18.2%) |
| 2020 | 18 of 159 (11.3%) |
| 2021 | 16 of 159 (10.1%) |
| 2022 | 25 of 159 (15.7%) |
| 2023 | 22 of 159 (13.8%) |
| 2024 | 9 of 159 (5.7%) |
| Below you will find questions to assess your nasal congestion prior to the surgery you had. Please rate the severity of your symptoms on scale from 0 to 4  *(Poniżej znajdują się pytania oceniające uczucie zatkanie nosa* sprzed zabiegu operacyjnego***,*** *który został u państwa wykonany. Proszę ocenić nasilenie objawów w skali od 0 do 4)* | |
| Nasal congestion or stuffiness ***(****Uczucie zatkanego/obrzękniętego nosa)* | |
| 0 | 9 of 159 (5.6%) |
| 1 | 16 of 159 (10.1%) |
| 2 | 36 of 159 (22.6%) |
| 3 | 61 of 159 (38.4%) |
| 4 | 37 of 159 (23.3%) |
| Nasal blockage or obstruction *(Obecność przeszkody/zwężenia w nosie)* | |
| 0 | 10 of 159 (6.3%) |
| 1 | 31 of 159 (19.5%) |
| 2 | 43 of 159 (27.0%) |
| 3 | 54 of 159 (34.0%) |
| 4 | 21 of 159 (13.2%) |
| Trouble breathing through my nose *(Trudności w oddychaniu przez nos)* | |
| 0 | 8 of 159 (5.0%) |
| 1 | 13 of 159 (8.2%) |
| 2 | 37 of 159 (23.3%) |
| 3 | 49 of 159 (30.8%) |
| 4 | 52 of 159 (32.7%) |
| Trouble sleeping *(Problemy ze snem)* | |
| 0 | 49 of 159 (30.8%) |
| 1 | 32 of 159 (20.1%) |
| 2 | 36 of 159 (22.6%) |
| 3 | 31 of 159 (19.5%) |
| 4 | 11 of 159 (6.9%) |
| Unable to get enough air through my nose during excercise or exertion  *(Trudności w ,,złapaniu oddechu" przez nos podczas wysiłku fizycznego lub po aktywności fizycznej)* | |
| 0 | 34 of 159 (21.4%) |
| 1 | 46 of 159 (28.9%) |
| 2 | 19 of 159 (11.9%) |
| 3 | 38 of 159 (23.9%) |
| 4 | 22 of 159 (13.8%) |
| Did you use intranasal oxy- and xylometazoline medications prior to septoplasty?  *(Czy używał/a Pan/Pani donosowych preparatów z oxy- i xylometazoliną przed operacją septoplastyki?)* | |
| Yes (*Tak)* | 104 of 159 (65,4%) |
| No *(Nie)* | 55 of 159 (34,6%) |
| Do you have a history of excessive use of intranasal oxy- and xylometazoline medications prior to septoplasty (i.e., using sprays daily for more than 7 days in a row)?  *(Czy zdarzyło się Panu/Pani nadużywać donosowych preparatów oxy- i xylometazoliny przed operacją septoplastyki (tj. Stosować krople codziennie dłużej niż 7 dni?))* | |
| Yes (*Tak)* | 52 of 104 (50%) |
| No *(Nie)* | 52 of 104 (50%) |
| What was the longest period of intranasal oxy- and xylometazoline medications overuse?  *(Jaki był najdłuższy okres nadużywania donosowych preparatów z oxy- i xylometazoliną?)* | |
| No longer than 2 weeks  *(Nie dłużej* *niż 2 tygodnie*) | 16 of 52 (30.8%) |
| Approximately one month  (*Około jednego miesiąca*) | 15 of 52 (28.8%) |
| Few months  (*Kilka miesięcy*) | 14 of 52 (26.9%) |
| Longer than a year  *(Dłuższy niż 1 rok)* | 7 of 52 (13.5%) |
| Since when have you periodically overuse intranasal oxy- and xylometazoline medications?  *(Od kiedy okresowo nadużywa Pan/Pani donosowych preparatów z oxy- i xylometazoliną?)* | |
| For about a year  (Od około roku) | 4 of 52 (7.7%) |
| Since about 2-3 years  *(Od około 2-3 lat)* | 26 of 52 (50%) |
| Since about 4-5 years  (*Od około 4-5* *lat)* | 17 of 52 (13.7%) |
| Since about 5-10 years  *(Od około 5*-*10 lat*) | 2 of 52 (3.8%) |
| Above 10 years  (*Powyżej 10 lat*) | 3 of 52 (5.8%) |
| What accounted for the reason for daily use of intranasal oxy- and xylometazoline medications?  *(Z czego wynikała konieczność codziennego stosowania donosowych preparatów z oxy- i xylometazoliną?)* | |
| Inability to function without oxy- and xylometazoline  (*Brak możliwości funkcjonowania bez preparatów*) | 5 of 52 (9.6%) |
| Only from urge to improve comfort  (*Jedynie z chęci poprawy komfortu*) | 32 of 52 (61.5%) |
| At first from the desire to improve comfort, and later from the inability to function without them  (*Na początku z chęci poprawy komfortu, później z braku możliwości funkcjonowania*) | 15 of 52 (28.8%) |
| Did you use steroid intranasal medications prior to septoplasty surgery?  *(Czy stosował/a Pan/Pani steroidowe krople donosowe przed operacją septoplastyki?)* | |
| No, I have not used them, but I knew there was such an option (*Nie, nie stosowałem/am ich, ale wiedziałem/am, że istnieje taka* *opcja*) | 8 of 52 (15.4%) |
| No, I have not used them, I did not know that there is such an option (*Nie, nie wiedziałem/am, że istnieje taka opcja*) | 16 of 52 (30.8%) |
| Yes, but their effect did not meet my expectations  (*Tak, ale ich efekt nie spełniał moich oczekiwań*) | 20 of 52 (38.5%) |
| Yes, their effect was satisfying to me  (*Tak, ich efekt był dla mnie zadowalający*) | 8 of 52 (15.4%) |
| Did you use nasal irrigation medications prior to septoplasty surgery?  *(Czy stosował/a Pan/Pani preparaty z solą fizjologiczną przed operacją septoplastyki?)* | |
| No, but I did know that there was such an option  (*Nie, ale wiedziałem/am, że istnieje taka* *opcja*) | 11 of 52 (21.2%) |
| Yes, but their effect did not meet my expectations  *(Tak, ale ich efekt nie spełniał moich oczekiwań*) | 25 of 52 (48.1%) |
| Yes, but their use is inconvenient  (*Tak, ale ich stosowanie jest uciążliwe*) | 4 of 52 (7.7%) |
| Yes, their effect was satisfying to me  (*Tak, ich efekt był dla mnie zadowalający)* | 12 of 52 (23.0%) |
| Were you aware of the adverse effects of intranasal oxy- and xylometazoline medications before surgery?  *(Czy miał/a Pan/Pani świadomość skutków niepożądanych przewlekłego stosowania preparatów oxy- i xylometazoliny przed operacją septoplastyki?)* | |
| Yes, I was aware  *(Miałem*) | 22 of 52 (42.3%) |
| I had, but only after using them for a period of time  *(Miałem, ale dopiero po pewnym czasie ich stosowania*) | 10 of 52 (19.2%) |
| No, I was not aware  (*Nie miałem*) | 20 of 52 (38.5%) |
| Where did you get your information on the adverse effects of chronic use of intranasal oxy- and xylometazoline medications?  *(Skąd uzyskał/a Pan/Pani informacje dotyczące niepożądanych skutków przewlekłego stosowania preparatów z oxy- i xylometazoliną?* | |
| From a doctor/pharmacist  (*Od lekarza/farmaceuty)* | 17 of 32 (53.1%) |
| From the internet  (*Z internetu*) | 10 of 32 (31.3%) |
| From the pharmaceutical leaflet  (*Z ulotki)* | 18 of 32 (56.3%) |
| From a friend/family member  (*Od znajomego/rodziny*) | 3 of 32 (9.4%) |
| Based on the adverse effects I have experienced  (*Na podstawie niekorzystnych skutków, które u mnie wystąpiły*) | 13 of 32 (40.6%) |
| From the television  (*Z telewizji*) | 1 of 32 (3.1%) |
| If you have used any other substances or treatments not mentioned above to improve nasal breathing comfort, please list them  *(Jeśli stosował/a Pan/Pani inne środki niż wyżej wymienione w celu poprawy komfortu oddychania przez nos proszę je wymienić)* | |
| Cirrus | 7 of 26 (26.9%) |
| Ibuprom sinus *(Ibuprom zatoki)* | 6 of 26 (23.1%) |
| Acatar | 3 of 26 (11.5%) |
| Sudafed | 2 of 26 (7.7%) |
| Pronasal | 1 of 26 (3.8%) |
| Hyaluronic acid inhalation  *(Inhalacje z kwasem hialuronowym)* | 1 of 26 (3.8%) |
| Herbal inhalations  *(Inhalacje ziołowe)* | 1 of 26 (3.8%) |
| Renopuren | 1 of 26 (3.8%) |
| I don’t remember  *(Nie pamiętam)* | 4 of 26 (15.4%) |
